# The A137R Protein of African Swine Fever Virus Inhibits Type I Interferon Production via the Autophagy-Mediated Lysosomal Degradation of TBK1

**DOI:** 10.1128/jvi.01957-21

**Published:** 2022-04-12

**Authors:** Maowen Sun, Shaoxiong Yu, Hailiang Ge, Tao Wang, Yongfeng Li, Pingping Zhou, Li Pan, Yu Han, Yuying Yang, Yuan Sun, Su Li, Lian-Feng Li, Hua-Ji Qiu

**Affiliations:** a State Key Laboratory of Veterinary Biotechnology, National African Swine Fever Para-Reference Laboratory, National High Containment Facilities for Animal Diseases Control and Prevention, Harbin Veterinary Research Institute, Chinese Academy of Agricultural Sciences, Harbin, China; b College of Animal Sciences, Yangtze University, Jingzhou, China; Hudson Institute of Medical Research

**Keywords:** African swine fever virus, A137R protein, IFN-β, TBK1, autophagy-mediated lysosomal pathway

## Abstract

African swine fever is a lethal hemorrhagic disease of pigs caused by African swine fever virus (ASFV), which greatly threatens the pig industry in many countries. Deletion of virulence-associated genes to develop live attenuated ASF vaccines is considered to be a promising strategy. A recent study has revealed that the *A137R* gene deletion results in ASFV attenuation, but the underlying mechanism remains unknown. To elucidate the mechanism of the *A137R* gene regulating ASFV virulence, an ASFV mutant with the *A137R* gene deleted (ASFV-ΔA137R) was generated based on the wild-type ASFV HLJ/2018 strain (ASFV-WT). Using transcriptome sequencing analysis, we found that ASFV-ΔA137R induced higher type I interferon (IFN) production in primary porcine alveolar macrophages (PAMs) than did ASFV-WT. Overexpression of the A137R protein (pA137R) inhibited the activation of IFN-β or IFN-stimulated response element. Mechanistically, pA137R interacts with TANK-binding kinase 1 (TBK1) and promotes the autophagy-mediated lysosomal degradation of TBK1, which blocks the nuclear translocation of interferon regulator factor 3, leading to decreased type I IFN production. Taken together, our findings clarify that pA137R negatively regulates the cGAS-STING-mediated IFN-β signaling pathway via the autophagy-mediated lysosomal degradation of TBK1, which highlights the involvement of pA137R regulating ASFV virulence.

**IMPORTANCE** African swine fever (ASF) is a lethal viral disease of pigs caused by African swine fever virus (ASFV). No commercial vaccines and antiviral treatments are available for the prevention and control of the disease. Several virulence-associated genes of ASFV have been identified, but the underlying attenuation mechanisms are not clear. Compared with the virulent parental ASFV, the *A137R* gene-deleted ASFV mutant promoted the expression of type I interferon (IFN) in primary porcine alveolar macrophages. Further analysis indicated that the A137R protein negatively regulated the cGAS-STING-mediated IFN-β signaling pathway through targeting TANK-binding kinase 1 (TBK1) for autophagy-mediated lysosomal degradation. This study not only facilitates the understanding of ASFV immunoevasion strategies, but also provides new clues to the development of live attenuated ASF vaccines.

## INTRODUCTION

African swine fever (ASF) is a highly contagious and hemorrhagic disease of pigs in many countries. From the first outbreak in Kenya in 1921, ASF has been prevalent in Europe, South America, and the Caribbean. The disease has caused significant economic losses due to its high mortality and wide spread since its emergence in China in 2018 (1–3). African swine fever virus (ASFV), the causative agent of ASF, belongs to the *Asfarviridae* family. As a nucleocytoplasmic large DNA virus, ASFV encodes more than 160 proteins, some of which are involved in viral DNA replication and repair, virion morphogenesis, and evasion of innate immune responses (4, 5).

Pathogen-associated molecular patterns (PAMPs) are sensed by the pathogen pattern recognition receptors (PRRs) to trigger the innate immune response. Upon binding to cytosolic double-stranded DNA (dsDNA), cyclic GMP-AMP (cGAMP) synthase (cGAS) recruits ATP and GTP to generate cGAMP. As a second messenger, cGAMP activates the cGAS-stimulator of interferon (IFN) genes (STING). Activated STING recruits TANK-binding kinase 1 (TBK1) to phosphorylate interferon regulator 3 (IRF3), leading to IFN-β production. Compared with the attenuated ASFV NH/P68 strain, the virulent Armenia/07 strain suppresses IFN-β production via the cGAS-STING pathway (6). It has been shown that the ability of ASFV to downregulate IFN production is associated with its virulence (7–11). So far, several ASFV proteins regulating IFN production have been identified. The ASFV A276R protein inhibits IFN-β production through targeting IRF3 (12); the I239L protein inhibits double-stranded RNA (dsRNA)-stimulated activation of IFN-β and CCL5, whereas the underlying mechanism remains to be elucidated (13); the DP96R protein negatively regulates type I IFN production by inhibiting the phosphorylation of TBK1 (14). The members of multigene families 360 (MGF360) and MGF505 are considered to be IFN antagonists (8–11, 15–17). The MGF360-12L protein disrupts the interaction between p65 and importin alpha to suppress the nuclear translocation of NF-κB (15), and the MGF505-11R protein degrades STING to antagonize type I IFN production via the lysosomal, ubiquitin-proteasome, and autophagy pathways (16). In addition, the MGF505-7R protein is involved in the pathogenicity of ASFV through targeting multiple adaptors of innate immune signaling pathways (9–11).

Most studies focus on IFN production antagonized by viral early-expressed proteins. However, the late-expressed viral proteins also block IFN production. The ASFV E120R protein directly interacts with IRF3 and disrupts the recruitment of IRF3 to TBK1, suppressing the activation of IRF3 (18). Interestingly, a series of late-expressed genes are also associated with ASFV virulence, such as *E184L* (19), *A137R* (20), *EP402R* (21, 22), and *I177L* (23). More specifically, an ASFV mutant with the *A137R* gene deleted is attenuated and able to confer protection against the challenge of the parental ASFV Georgia2010 (ASFV-G) (20). However, the mechanism of the A137R protein (pA137R or p11.5) regulation of ASFV virulence remains unclear. Therefore, we analyzed the differentially expressed genes (DEGs) of the primary porcine alveolar macrophages (PAMs) infected with the wild-type ASFV HLJ/2018 strain (ASFV-WT) sharing a high identity with ASFV-G or the *A137R* gene-deleted ASFV mutant (ASFV-ΔA137R) by transcriptome sequencing (RNA-seq) analysis. Compared with ASFV-WT, ASFV-ΔA137R infection significantly increased type I IFN production in PAMs. Mechanistically, pA137R inhibits the cGAS-STING-mediated IFN-β signaling pathway by interacting with and degrading TBK1 in a dose-dependent manner via the autophagy-mediated lysosomal pathway, leading to the inhibition of IRF3 nuclear translocation. Altogether, our results dissect for the first time the underlying mechanism of pA137R suppressing type I IFN production, which is associated with ASFV virulence.

## RESULTS

### ASFV-ΔA137R induces higher type I IFN production in PAMs than does ASFV-WT.

**FIG 1:**
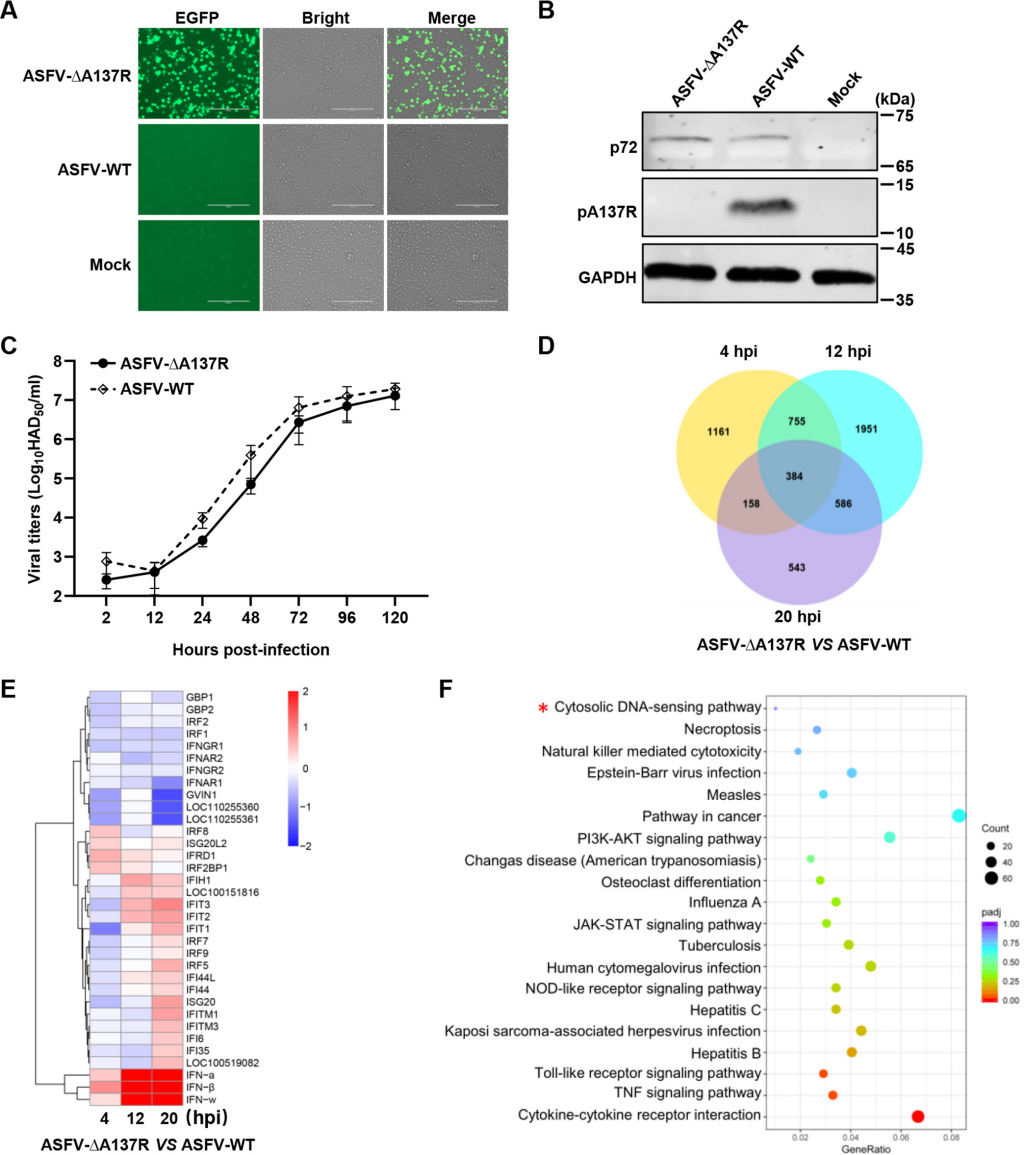
Differential expression pattern in *A137R* gene-deleted ASFV mutant (ASFV-ΔA137R)-infected primary porcine alveolar macrophages (PAMs) by transcriptome sequencing (RNA-seq) analysis. (A and B) Identification of ASFV-ΔA137R by fluorescence microscopy (A) and Western blotting (B). (C) Replication characteristics of ASFV-ΔA137R and the wild type ASFV HLJ/2018 strain (ASFV-WT) in PAMs. PAMs were infected with ASFV-ΔA137R or ASFV-WT at a multiplicity of infection (MOI) of 0.01 and collected at the indicated time points to determine the viral titers by 50% hemadsorption doses (HAD_50_). (D) Venn diagram of the differentially expressed genes (DEGs) in the ASFV-ΔA137R- versus ASFV-WT-infected PAMs. PAMs were infected with ASFV-ΔA137R or ASFV-WT (MOI = 1) and collected at 4, 12, and 20 hours post-infection (hpi), and then the total mRNAs of PAMs were used to generate RNA-seq libraries. A total of 384 DEGs (|log_2_ fold change| of >1) were expressed in the three groups: the numbers of special genes from ASFV-ΔA137R versus ASFV-WT at 4, 12, and 20 hpi are 1,161, 1,951, and 543, respectively. (E) Heat map of the 34 DEGs involved in IFN response induced by ASFV-ΔA137R versus ASFV-WT at 4, 12, and 20 hpi. (F) Kyoto Encyclopedia of Genes and Genomes enrichment analysis in the ASFV-ΔA137R- or ASFV-WT-infected PAMs. Error bars denote standard errors of the means. A red asterisk indicates the signaling pathway of interest.

To explore the underlying molecular mechanism of pA137R regulation of ASFV virulence, we generated ASFV-ΔA137R based on the virulent ASFV HLJ/2018 strain by homologous recombination. The *A137R* gene was replaced by a cassette containing the enhanced green fluorescent protein (*EGFP*) gene under the control of the ASFV *p72* promoter (*p72*EGFP). ASFV-ΔA137R was purified after 10 rounds of limiting dilution based on the EGFP expression (Fig. 1A), and the purified mutant virus was identified by Western blotting (Fig. 1B). As expected, pA137R was detectable in the PAMs infected with ASFV-WT, but not with ASFV-ΔA137R, while p72 could be detected in the PAMs infected with either ASFV-ΔA137R or ASFV-WT. To determine the expected deletion of the *A137R* gene, the genome of ASFV-ΔA137R was analyzed by next-generation sequencing (NGS). Compared with the parental ASFV-WT, no undesirable variations were found in the genome of ASFV-ΔA137R, except a 391-bp deletion of the *A137R* gene replaced by the *p72*EGFP expression cassette. We then analyzed the effect of the *A137R* gene deletion on viral growth *in vitro*. PAMs were infected with ASFV-ΔA137R or ASFV-WT at a multiplicity of infection (MOI) of 0.01, and the viral titers were assayed by 50% hemadsorption doses (HAD_50_) at the indicated time points. The growth profile of ASFV-ΔA137R was similar to that of ASFV-WT in PAMs (Fig. 1C).

The virulence of ASFV depends on the viral replication efficiency in target cells or the blockage of the immune responses by viral proteins. Since the *A137R* gene deletion does not influence the replication of ASFV-WT *in vitro*, we thus systemically analyzed the DEGs of the PAMs infected with ASFV-ΔA137R or ASFV-WT (MOI = 1) by RNA-seq analysis. Compared with the ASFV-WT-infected PAMs, the transcription levels of 2,458, 3,676, and 1,671 genes were altered upon ASFV-ΔA137R infection at 4, 12, and 20 hours post-infection (hpi), respectively (Fig. 1D). Notably, ASFV-ΔA137R infection induced higher type I IFN production in PAMs at 12 and 20 hpi, including *IFN*-ɑ, *IFN*-β, and *IFN*-ω, than did ASFV-WT (Fig. 1E). The Kyoto Encyclopedia of Genes and Genomes (KEGG) enrichment analysis indicated that the upregulated genes upon ASFV-ΔA137R infection were involved in the IFN signaling pathway, such as cytosolic DNA-sensing, JAK-STAT, and Toll-like receptor (TLR) signaling pathways (Fig. 1F).

**FIG 2:**
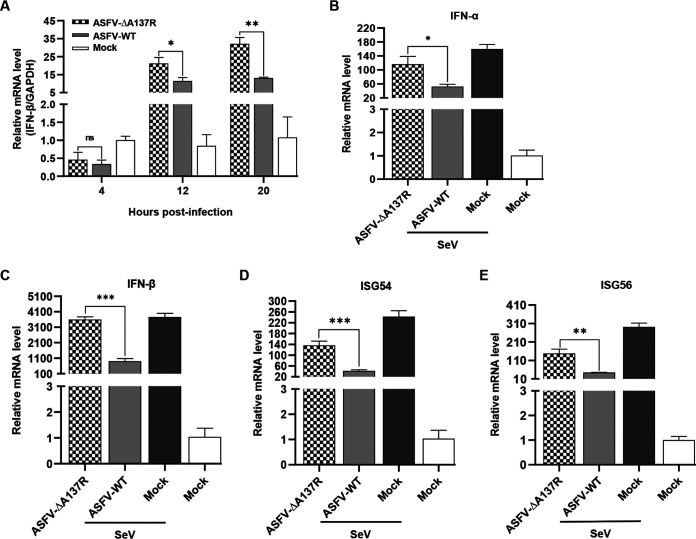
The *A137R* gene-deleted ASFV mutant (ASFV-ΔA137R) induces higher type I interferon (IFN) production in primary porcine alveolar macrophages (PAMs) than does the wild-type ASFV (ASFV-WT). (A) ASFV-ΔA137R promoted IFN-β production. PAMs were infected with ASFV-ΔA137R or ASFV-WT, and the mRNA level of *IFN*-*β* was quantified by reverse transcription-quantitative PCR (RT-qPCR) at 4, 12, and 20 hours post-infection. (B to E) pA137R inhibits the production of type I IFNs and IFN-stimulated genes (ISGs) induced by Sendai virus (SeV). PAMs were infected with ASFV-ΔA137R or ASFV-WT for 24 h at a multiplicity of infection of 1 and then stimulated with SeV for 12 h to examine the mRNA level of *IFN*-*α* (B), *IFN*-*β* (C), *ISG54* (D), or *ISG56* (E) by RT-qPCR. Error bars denote standard errors of the means. All the data were analyzed using Student's *t* test: *, *P < *0.05; **, *P < *0.01; ***, *P < *0.001.

To verify the roles of pA137R regulation of the IFN signaling pathway, the transcription level of *IFN-β* was examined by reverse transcription-quantitative PCR (RT-qPCR) in the ASFV-ΔA137R- or ASFV-WT-infected PAMs. As shown in Fig. 2A, ASFV-ΔA137R, but not ASFV-WT, significantly enhanced *IFN-β* transcription in the PAMs at 12 to 20 hpi but not at 4 hpi, which was consistent with the RNA-seq analysis. Sendai virus (SeV), an IFN agonist, activates the retinoic acid inducible gene (RIG-I)-mediated IFN signaling pathway (24). ASFV-ΔA137R also induced higher transcription levels of *IFN*-ɑ, *IFN*-β, and downstream IFN-stimulated genes (ISGs), including *ISG54* and *ISG56*, in the PAMs upon SeV stimulation than did ASFV-WT (Fig. 2B to E). Taken together, these data indicate that pA137R inhibits the IFN signaling pathway and plays an important role in evading innate immune response.

### pA137R inhibits the cGAS-STING-mediated IFN-β signaling pathway.

**FIG 3:**
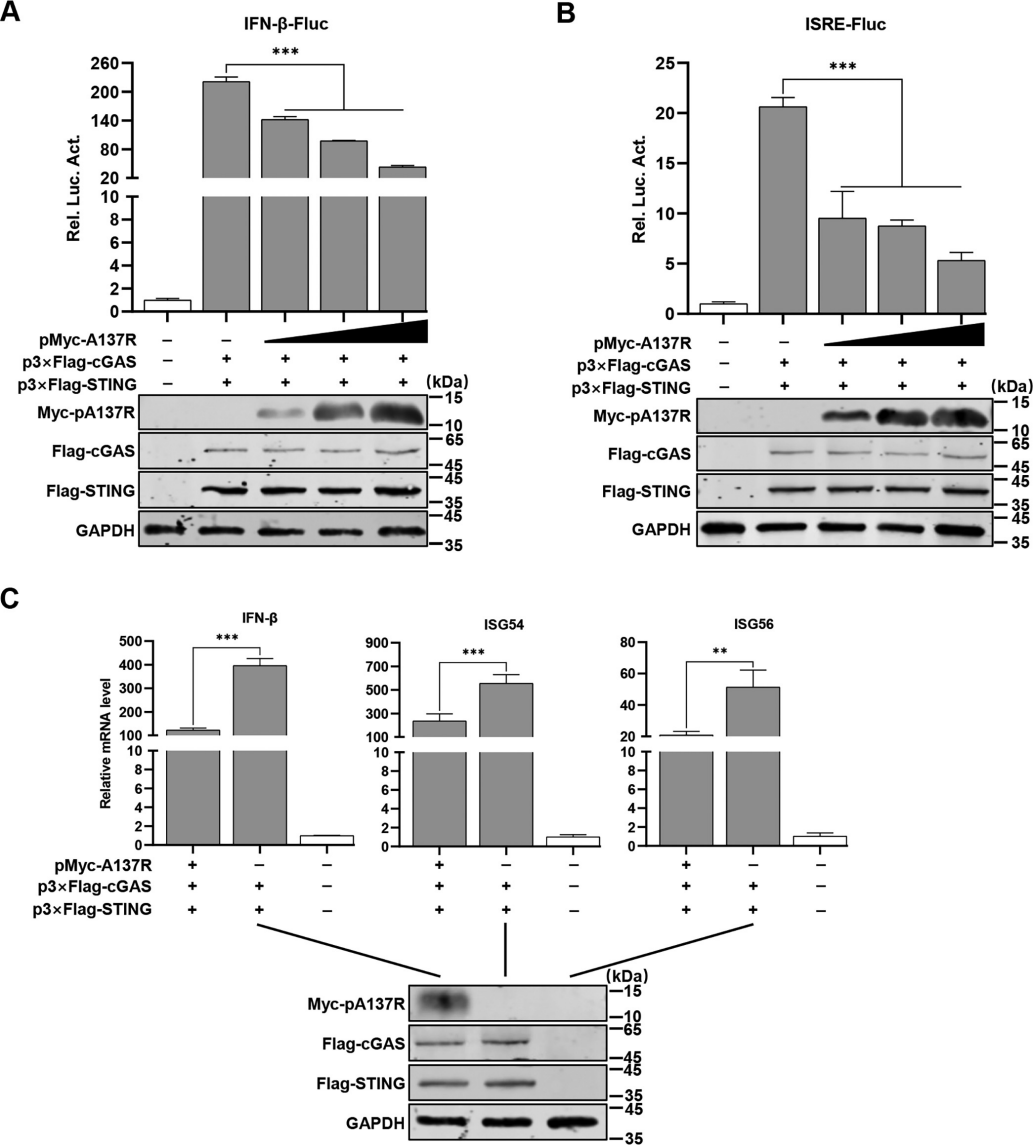
The A137R protein (pA137R) inhibits the cGAS-STING-mediated beta interferon (IFN-β) signaling pathway. (A and B) pA137R inhibits the activation of the IFN-β (A) or IFN-stimulated response element (ISRE) promoter (B) in a dose-dependent manner. HEK293T cells were cotransfected with pIFN-β/pISRE-Fluc expressing firefly luciferase (0.05 μg), pTK-Rluc expressing *Renilla* luciferase (0.01 μg), and p3×Flag-cGAS (0.01 μg) and -STING (0.05 μg), along with pMyc-A137R (0.1, 0.25, or 0.5 μg) for 24 h followed by luciferase reporter assay. The expression of cGAS, STING, or pA137R was analyzed by Western blotting using mouse anti-Flag or -Myc monoclonal antibody. GAPDH was used as a loading control. (C) pA137R inhibits the mRNA levels of *IFN*-*β* and *IFN*-stimulated genes (ISGs). HEK293T cells were cotransfected with p3×Flag-cGAS and -STING along with pMyc-A137R (0.5 μg) or pCMV-Myc (0.5 μg) for 24 h, and the mRNA levels of *IFN*-*β*, *ISG54*, and *ISG56* were quantified by reverse transcription-quantitative PCR. Error bars denote standard errors of the means. All the data were analyzed using Student's *t* test: **, *P < *0.01; ***, *P < *0.001.

The cGAS-STING-mediated IFN-β signaling pathway is significantly suppressed upon the virulent ASFV Armenia/07 infection, but the viral proteins involved remain elusive (5). The RNA-seq analysis revealed that the cytosolic DNA-sensing signaling pathway was activated in the ASFV-ΔA137R-infected PAMs, and thus we further investigated the effects of pA137R on the cGAS-STING pathway. Human embryonic kidney (HEK293T) cells were cotransfected with p3×Flag-cGAS and -STING and reporter plasmids, along with pMyc-A137R or pCMV-Myc, for 24 h. The results indicated that overexpression of pA137R significantly inhibited the activation of the IFN-β or IFN-stimulated response element (ISRE) promoter in a dose-dependent manner (Fig. 3A and B). Similarly, pA137R also markedly suppressed the transcription levels of *IFN*-*β*, *ISG54*, and *ISG56* (Fig. 3C). These data suggest that pA137R negatively regulates the cGAS-STING-mediated IFN-β signaling pathway.

### pA137R inhibits the IFN-β signaling pathway through targeting TBK1.

**FIG 4:**
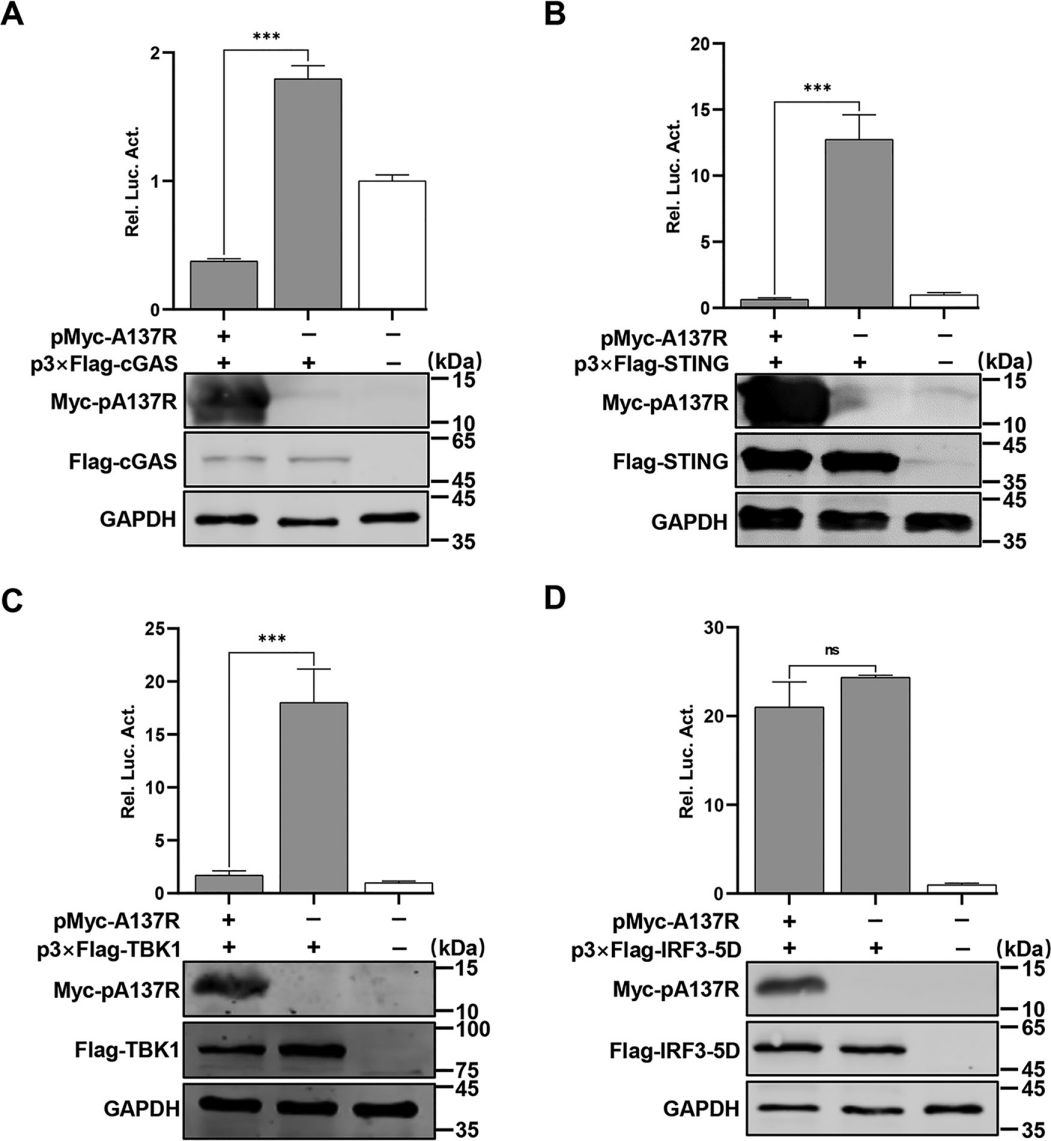
The A137R protein (pA137R) inhibits the beta interferon (IFN-β) signaling pathway through targeting TBK1. (A to D) HEK293T cells were cotransfected with pIFN-β-Fluc expressing firefly luciferase (0.05 μg), pTK-Rluc expressing *Renilla* luciferase (0.01 μg), and p3×Flag-cGAS (A), -STING (B), -TBK1 (C), or -IRF3-5D (the activated form of IRF3) (D), along with pMyc-A137R or pCMV-Myc, and the luciferase activities were measured at 24 hours post-infection. The expression of cGAS, STING, TBK1, IRF3-5D, or pA137R was analyzed by Western blotting using mouse anti-Flag or -Myc monoclonal antibody. GAPDH was used as a loading control. Error bars denote standard errors of the means. All the data were analyzed using Student's *t* test: ***, *P < *0.001; ns, not significant.

**FIG 5:**
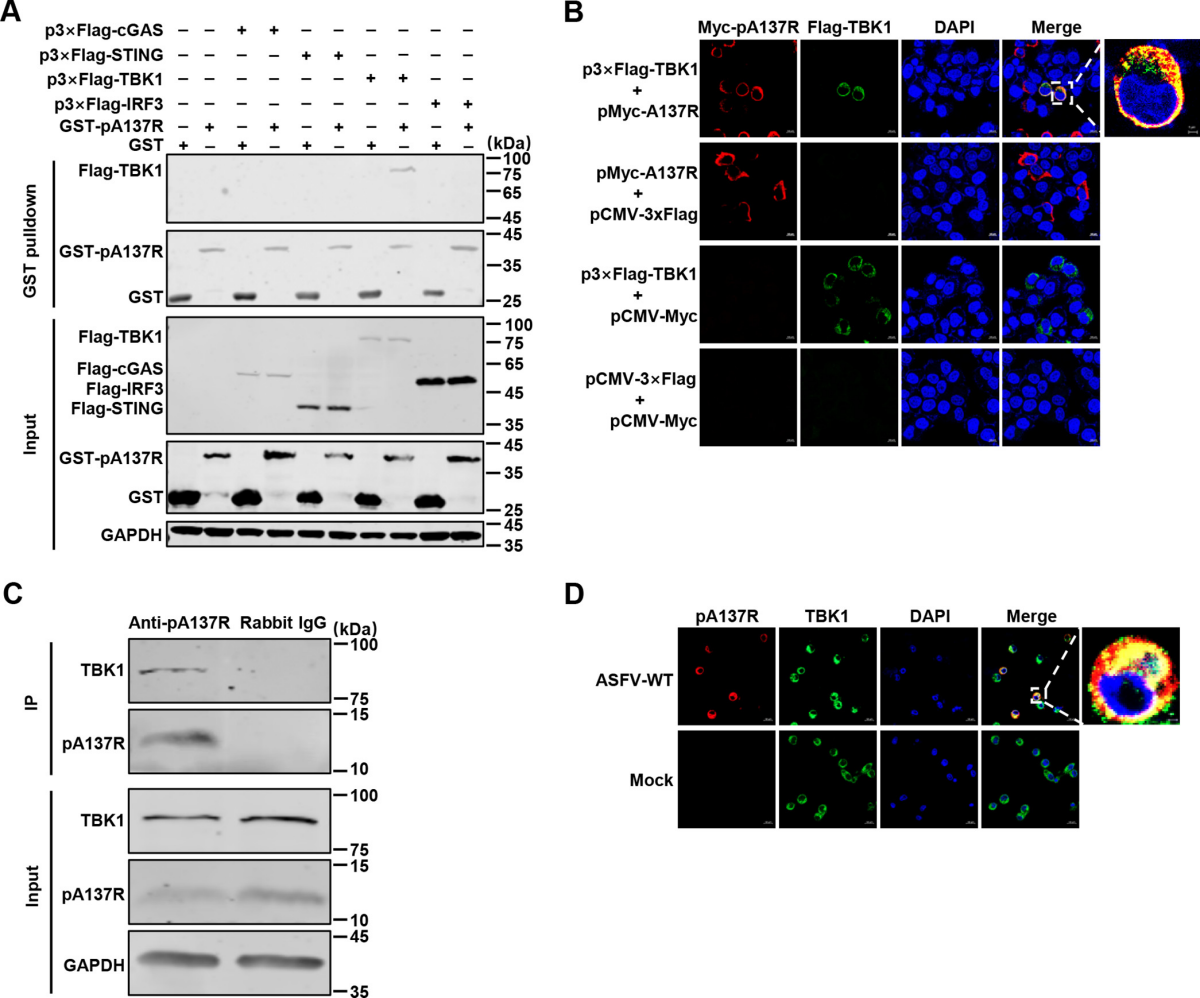
The A137R protein (pA137R) interacts with TBK1. (A) HEK293T cells were transfected with p3×Flag-cGAS, -STING, -TBK1, or -IRF3 for 48 h and lysed with NP-40 buffer. The purified GST or GST-pA137R protein was used to pull down the key adaptors of the cGAS-STING pathway in the lysates and analyzed by Western blotting using mouse anti-GST or -Flag monoclonal antibody (MAb). (B) HEK293T cells were cotransfected with p3×Flag-TBK1 and pMyc-A137R for 24 h and then incubated with rabbit anti-Flag or mouse anti-Myc MAb and Alexa Fluor 488 (green)- or 633 (red)-conjugated secondary antibody, respectively. Cell nuclei (blue) were stained with 4′,6-diamidino-2-phenylindole (DAPI). Bars, 10 μm. (C) Primary porcine alveolar macrophages (PAMs) were infected with the wild-type ASFV HLJ/2018 strain (ASFV-WT) at a multiplicity of infection (MOI) of 1 for a coimmunoprecipitation assay. The lysates were collected at 48 hours post-infection and incubated with protein G agarose, along with rabbit anti-pA137R polyclonal antibodies (PAbs) or irrelevant rabbit immunoglobulin G (IgG), and then the bound proteins were analyzed by Western blotting using in-house mouse anti-pA137R or rabbit anti-TBK1 PAbs. (D) PAMs were uninfected or infected with ASFV-WT for 24 h at an MOI of 1, and the localization of pA137R and TBK1 was visualized by laser confocal microscopy using in-house mouse anti-pA137R or rabbit anti-TBK1 PAbs and the indicated secondary antibody, respectively. Cell nuclei (blue) were stained with DAPI as described above. Bars, 10 μm.

To identify which adaptors in the cGAS-STING pathway were antagonized by pA137R, HEK293T cells were cotransfected with pMyc-A137R and reporter plasmids along with p3×Flag-cGAS, -STING, -TBK1, or -IRF3-5D (the active form of IRF3), respectively. We showed that pA137R markedly suppressed the activation of the IFN-β promoter induced by cGAS, STING, or TBK1, but not IRF3-5D (Fig. 4A to D). Therefore, we speculated that pA137R may inhibit the activation of the IFN-β promoter through targeting TBK1. Glutathione *S*-transferase (GST) pulldown assay indicated that pA137R specifically interacted with TBK1 but not with other adaptors, including cGAS, STING, or IRF3, of the cGAS-STING pathway (Fig. 5A). The colocalization of pA137R with TBK1 was observed in the cytoplasm, with a colocalization coefficient of 0.74 (Fig. 5B). PAMs were infected with ASFV-WT for 48 h, and the endogenous TBK1 was subjected to coimmunoprecipitation (co-IP) assay using anti-pA137R polyclonal antibodies (PAbs). As shown in Fig. 5C, TBK1 was precipitated by pA137R in the ASFV-infected PAMs. The colocalization of pA137R and TBK1 (a colocalization coefficient of 0.75) was further demonstrated by laser confocal microscopy (Fig. 5D). Collectively, these results indicate that pA137R blocks the IFN-β signaling pathway by interacting with TBK1.

### pA137R promotes the autophagy-mediated lysosomal degradation of TBK1.

**FIG 6:**
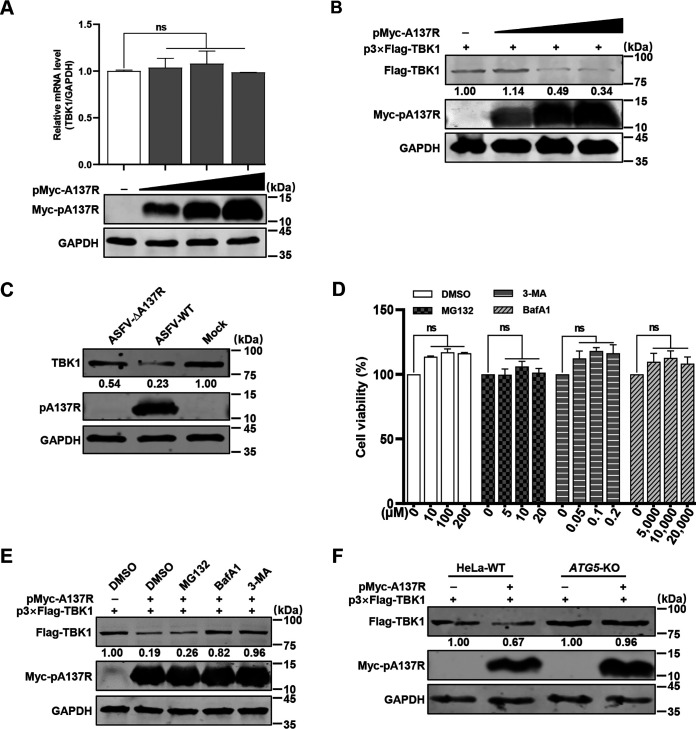
The A137R protein (pA137R) promotes the autophagy-mediated lysosomal degradation of TBK1. (A and B) pA137R decreases TBK1 expression. (A) HEK293T cells were transfected with pMyc-A137R (0, 0.25, 0.5, or 1 μg), and subjected to isolation of cellular total mRNAs followed by quantification of TBK1 transcription level by reverse transcription-quantitative PCR. (B) HEK293T cells were cotransfected with p3’Flag-TBK1 (1 μg) together with pMyc-A137R (0, 0.25, 0.5, or 1 μg), and subjected to isolation of total cellular proteins followed by analysis by Western blotting. (C) The *A137R* gene-deleted ASFV mutant (ASFV-ΔA137R) resulted in higher TBK1 expression in primary porcine alveolar macrophages (PAMs) than did the wild-type ASFV HLJ/18 strain (ASFV-WT). PAMs were infected with ASFV-ΔA137R or ASFV-WT for 24 hours post-infection (hpi) at a multiplicity of infection (MOI) of 1, and the expression of TBK1 or pA137R was analyzed by Western blotting using the indicated antibodies. (D) Viability of HEK293T cells treated with the indicated inhibitors. HEK293T cells were grown for 24 h and then treated with dimethyl sulfoxide (DMSO [negative control]) (0, 10, 100, or 200 μM), MG132 (0, 5, 10, or 20 *μ*M), bafilomycin A1 (BafA1) (0, 50, 100, or 200 nM), or 3-methyladenine (3-MA) (0, 5, 10, or 20 mM) for 8 h. The cell viability was determined as described above. (E) HEK293T cells were cotransfected with p3×Flag-TBK1 and pMyc-A137R or pCMV-Myc for 24 h and then treated for 8 h with DMSO (negative control), MG132 (20 μM), BafA1 (100 nM), or 3-MA (10 mM), respectively. The expression of TBK1 or pA137R was analyzed as described above. GAPDH was used as a loading control. (F) pMyc-A137R and p3×Flag-TBK1 were cotransfected into autophagy-related protein 5 (*ATG5*)-knockout or wild-type (WT) HeLa cells for 36 h, and the expression of TBK1 or pA137R was examined with the indicated antibodies as described above. GAPDH was used as a loading control. Error bars denote standard errors of the means. All the data were analyzed using Student's *t* test. ns, not significant.

Previous studies have revealed that the IFN signaling pathway is antagonized by several viruses, including human cytomegalovirus (HCMV) (25), Zika virus (ZIKV) (26), and severe acute respiratory syndrome coronavirus 2 (SARS-CoV-2) (27), through targeting or degrading the host proteins. Since pA137R interacts with TBK1 and inhibits the activation of IFN-β (Fig. 3 to 5), we investigated the effects of pA137R on the transcription and expression of TBK1. p3×Flag-TBK1 and pMyc-A137R were cotransfected into HEK293T cells. The results showed that TBK1 protein expression was inhibited by overexpression of pA137R in a dose-dependent manner, while the mRNA level of *TBK1* remained unchanged (Fig. 6A and B), indicating that pA137R may degrade the TBK1 protein. We further analyzed the TBK1 expression in the PAMs infected with ASFV-ΔA137R or ASFV-WT at 24 hpi. We found that ASFV-WT downregulated the expression of TBK1, while the TBK1 expression was significantly increased upon ASFV-ΔA137R infection in PAMs, although it was lower than that in the uninfected PAMs (Fig. 6C). Degradation of cellular proteins mainly relies on the ubiquitin-proteasome, autophagosome, or lysosome pathway. Therefore, HEK293T cells were cotransfected with p3×Flag-TBK1 and pMyc-A137R for 24 h and then treated with proteasome inhibitor MG132, autophagosome inhibitor 3-methyladenine (3-MA), or lysosomal inhibitor bafilomycin A1 (BafA1), respectively, for 8 h. None of the inhibitors affected the viability of HEK293T cells (Fig. 6D), and the pA137R-mediated TBK1 degradation was inhibited by BafA1 or 3-MA, but not MG132 (Fig. 6E). Since autophagy-related protein 5 (ATG5) is essential for autophagosome formation (28), we therefore examined the involvement of autophagosome in the degradation of TBK1 by pA137R using the *ATG5*-knockout HeLa cells. Consistent with the inhibitor treatment assay, the degradation of TBK1 by pA137R was counteracted in the *ATG5*-knockout HeLa cells compared with the parental wild-type (WT) cells (Fig. 6F). Altogether, these results indicate that the autophagy-mediated lysosomal pathway is responsible for the degradation of TBK1 by pA137R.

### pA137R blocks the nuclear translocation of IRF3.

**FIG 7:**
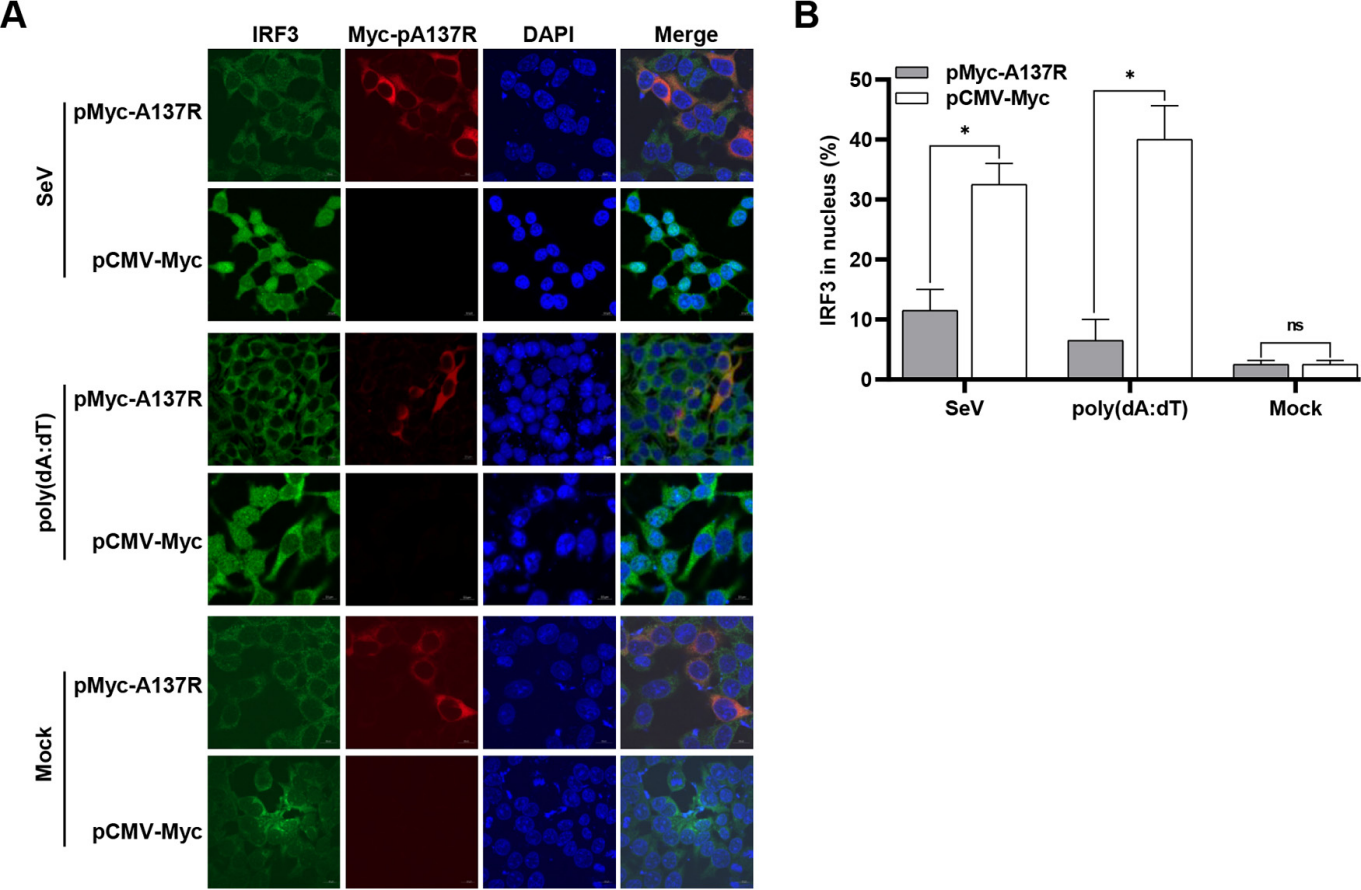
The A137R protein (pA137R) blocks the nuclear translocation of interferon factor 3 (IRF3). HEK293T cells were transfected with pMyc-A137R or pCMV-Myc for 24 h and then stimulated with Sendai virus (SeV) at a multiplicity of infection of 1 or transfected with poly(dA-dT) (2 μg/mL) for 8 h. (A) The subcellular localization of IRF3 (green), pA137R (red), or cell nuclei (blue) was observed by laser confocal microscopy. Bars, 10 μm. (B) The transfected cells with IRF3 nuclear translocation were counted from 100 cells per condition from different fields in panel A. Error bars denote standard errors of the means. All the data were analyzed using Student's *t* test: *, *P < *0.05; ns, not significant.

With the activation of the cGAS-STING pathway, the activated IRF3 is translocated into the nucleus and triggers *IFN*-β transcription. Since IRF3 is activated by TBK1, we speculated that TBK1 degradation may influence IRF3 translocation from the cytoplasm into the nucleus. HEK293T cells were transfected with pMyc-A137R or pCMV-Myc for 24 h and then stimulated with SeV or poly(dA-dT). The subcellular localization of IRF3 in the absence or presence of pA137R was determined by laser confocal microscopy (Fig. 7A and B). In the unstimulated group, the IRF3 was mainly located in the cytoplasm. Upon SeV or poly(dA-dT) stimulation, approximately 30 to 40% of IRF3 translocated into the nucleus in the pCMV-Myc-transfected cells. However, only 10% of IRF3 was detected in the nucleus when there was ectopic expression of pA137R. Thus, pA137R degrades TBK1, leading to blockage of the nuclear translocation of IRF3.

## DISCUSSION

**FIG 8:**
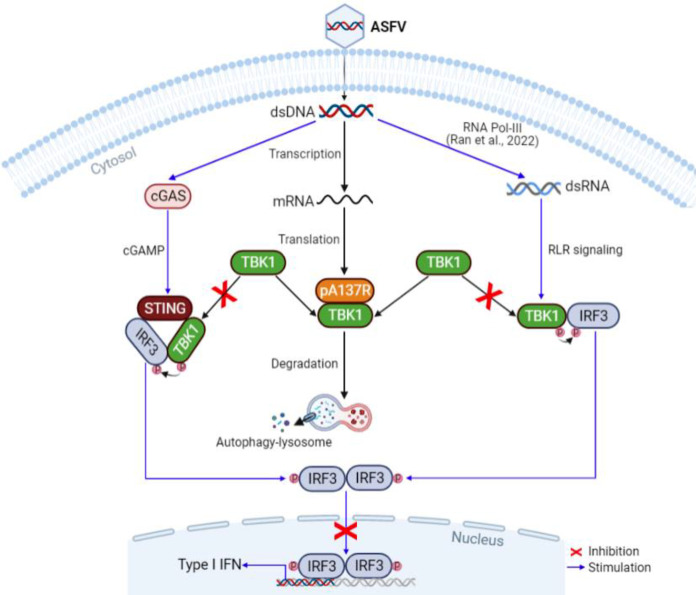
Schematic model of the A137R protein (pA137R) negatively regulating beta interferon (IFN-β) production upon ASFV infection. ASFV dsDNA can be sensed by cGAS, a cytosolic DNA sensor, and transcribed into dsRNA by type III RNA polymerase (RNA Pol-III) (47) to trigger the activation of the IFN signaling pathway. To evade innate immune responses, pA137R interacts with and degrades TBK1 via the autophagy-mediated lysosomal pathway, followed by the inhibition of IRF3 nuclear translocation to suppress the IFN-β production upon ASFV infection.

Previous studies have shown that knocking out the immunoregulatory genes usually alters ASFV virulence (8–11, 17, 29–31). pA137R is a late-expressed viral structural protein and associated with ASFV virulence (20, 32), but the underlying attenuation mechanism is not clear. In this study, we found that ASFV-ΔA137R significantly increased type I IFN production in PAMs compared with ASFV-WT (Fig. 1 and 2). pA137R suppressed the cGAS-STING-mediated IFN-β signaling pathway through targeting TBK1 (Fig. 3 and 4). Furthermore, we demonstrated that pA137R interacts with TBK1, promotes its degradation via the autophagy-mediated lysosomal pathway, and affects IRF3 nuclear translocation to block type I IFN production (Fig. 8). To our knowledge, this is the first study to elaborate the mechanism of pA137R regulation of ASFV virulence.

TBK1 undergoes autophosphorylation and ubiquitination to facilitate the activation of downstream adaptors (e.g., IRF3 or NF-κB) during viral infections. However, viral proteins target TBK1 to downregulate IFN production by disrupting TBK1-associated complexes or degrading TBK1: e.g., the HCMV UL94 protein (25), the herpes simplex virus 1 (HSV-1) US11 (33) and *γ*34.5 (34) proteins, the foot-and-mouth disease virus L^pro^ protein (35), the swine acute diarrhea syndrome coronavirus nucleocapsid protein (36), the heartland virus nonstructural proteins (37), and the SARS-CoV-2 M protein (38). In this study, we found that type I IFN production was inhibited by pA137R in PAMs upon ASFV infection (Fig. 1 and 2), and the TBK1 expression was also downregulated in the HEK293T cells with pA137R-overexpressed or ASFV-WT-infected PAMs (Fig. 6). TBK1, a critical adaptor of the cGAS-, RIG-I-, and melanoma differentiation-associated antigen 5 (MDA-5)-mediated signaling pathways (39), is degraded by ubiquitin-specific protease 38 (USP38) (40), major vault protein (MVP) (41), and dual-specificity tyrosine-(Y)-phosphorylation-regulated kinase 2 (DYRK2) (42) via the proteasomal or autophagy-mediated lysosomal pathway. It has been revealed that the ASFV DP96R protein degrades TBK1 (14), but the underlying mechanism remains unknown. We have demonstrated that pA137R interacts with and degrades TBK1 via the autophagy-mediated lysosomal pathway (Fig. 5 and 6). The expression of TBK1 in the ASFV-ΔA137R-infected PAMs was higher than that in the ASFV-WT-infected PAMs but lower than that in the uninfected PAMs, implying that other ASFV proteins besides pA137R might also degrade TBK1.

Vaccinia virus (VACV) replicates in the cytoplasm and generates dsRNA (43). The A52 (44), K7 (45), and E3 (46) proteins of VACV inhibit the TLR-mediated NF-κB activation. Similarly, ASFV (13, 47, 48) and HSV-1 (49, 50) also regulate the dsDNA or dsRNA-mediated antiviral responses. In this study, we found that ASFV-ΔA137R induced higher transcription of the *IFN* genes and ISGs in the PAMs upon SeV stimulation than did ASFV-WT, and pA137R negatively regulated the cGAS-STING-mediated IFN-β signaling pathway (Fig. 2 and 3), which indicated that pA137R is an IFN antagonist in the dsDNA (cGAS)- or dsRNA (RIG-I or MDA-5)-mediated IFN-β signaling pathway. RNA-seq analysis demonstrated that the JAK-STAT and TLR signaling pathways were activated, and the expression of chemokines (e.g., CCL2, CCL8, and CXCL8) and proinflammatory cytokines (e.g., interleukin-1α [IL-1α] and IL-1β) was upregulated in the ASFV-ΔA137R-infected PAMs at 12 to 20 hpi, implying that pA137R probably exerts multiple actions to evade the innate immune responses.

It is generally accepted that the early-expressed viral proteins play a key role in regulating IFN production, but several studies have shown that the late-expressed viral proteins are also involved in this process and associated with viral virulence. UL94, a late-expressed protein and structural component of HCMV, inhibits IFN-β production by disrupting the dimerization of MITA and recruitment of TBK1 to MITA (25, 51, 52). The HSV-1 US11 protein interacts with RIG-I and MDA-5 and antagonizes IFN-β production (50); the herpesviral *γ*34.5 protein prevents protein synthesis via the protein kinase R (PKR) signaling pathway or inhibits the production of IFN-β by promoting TBK1 degradation and disrupting the interaction between TBK1 and IRF3 (33, 53). IFN-β inhibits viral infections, and low-virulence viruses in turn increase IFN-β production, such as West Nile virus (54), Japanese encephalitis virus (55), bluetongue virus (56), and ASFV (6–11). Deletion of the IFN antagonist from the virulent ASFV results in attenuation (7–11). The *A137R* gene is associated with the virulence of ASFV (20), its deletion resulted in more production of IFN-α, IFN-β, ISG54, and ISG56 in PAMs (Fig. 1 and 2).

There is an urgent need to define the determinants of ASFV virulence, which contributes to the development of treatments and vaccines. A low dose (10^2^ HAD_50_) of the *A137R* gene-deleted ASFV mutant (ASFV-G-ΔA137R) results in ASFV attenuation and offers protection against challenge with the virulent parental ASFV-G, but the ASFV-G-ΔA137R-inoculated pigs present high viremia titers (10^5^ to 10^6^) and shed the virus (20). It has been reported that pA137R is highly conserved among the genotype I and II strains (20). Because the attenuation of ASFV is relevant to the increased production of IFN (7–11) and ASFV-ΔA137R promotes IFN-β production in PAMs, we speculate that pA137R promotes the autophagy-mediated lysosomal degradation of TBK1 to negatively regulate IFN-β production and attenuate ASFV virulence. However, we cannot rule out the differences in the genetic backgrounds of virus strains, which lead to changes in phenotypes. Deletion of *EP402R* from the virulent BA71 strain results in attenuation, whereas the phenotype is not observed in the ASFV Georgia 2007 and HLJ/2018 strains (22, 57–59). Deletion of *DP148R* from the Benin 97/1 strain leads to ASFV attenuation (60), but not the ASFV HLJ/2018 strain (59), which was isolated in China (58). However, the *UK*/*CD2v*-deleted ASFV strain also presents different attenuation in these strains (59, 61).

In conclusion, our research is the first to reveal that pA137R interacts with and degrades TBK1 via the autophagy-mediated lysosomal pathway to inhibit IFN-β production. These findings enrich the understanding of immunoevasion by ASFV and the mechanism of pA137R regulation of ASFV virulence.

## MATERIALS AND METHODS

### Cells and virus strain.

PAMs were cultured in RPMI 1640 medium (catalog no. C11875500BT; Gibco) supplemented with 10% fetal bovine sera (FBS) (catalog no. 10091148; Gibco) and 2% antibiotics-antimycotics (catalog no. 15140122; Gibco) (62). HEK293T (62), *ATG5*-knockout HeLa (63), or HeLa (64) cells were cultured in Dulbecco’s modified Eagle’s medium (DMEM) (catalog no. D6429; Sigma-Aldrich) with 10% FBS at 37°C with 5% CO_2_. The ASFV HLJ/2018 strain (GenBank no. MK333180.1) (58) and SeV (65) were propagated as described previously.

### Construction of plasmids.

The *A137R* gene was amplified from the genomic DNA (gDNA) of ASFV-WT and cloned into the EcoRI/XhoI sites of the pCMV-Myc (Clontech) or pGEX-6p-1 (GE Healthcare) vector, creating pMyc-A137R or pGST-A137R, respectively. The porcine *cGAS*, *STING*, *TBK1*, or *IRF3* gene was amplified from the cDNA of PK-15 cells and cloned into the p3×Flag-CMV-10 vector (Sigma-Aldrich) to generate p3×Flag-cGAS, -STING, -TBK1, or -IRF3, respectively. p3×Flag-IRF3-5D harbors an active form of IRF3 with five site-directed mutations (S394A, S396A, S400A, S403A, and T405A). The promoter reporter plasmids (pIFN-β-Fluc or pISRE-Fluc) expressing the firefly luciferase (Fluc) and the internal reference reporter plasmid pTK-Rluc expressing the *Renilla* luciferase (Rluc) were described previously (65). The primers for amplification of plasmids are listed in Table 1.

**TABLE 1:** Primers used in this study

Primers^*a*^	Sequence (5′→3′)	Description
dA137R-LA-F	GGAGCTCGAATTCGAAGCTTATCTGCAATTCAAAATCATTTAC	For left arm
dA137R-LA-R	CATCTCTCACGAGATCGTGACTGTACAGTTGTATACATATAAATTC	
72EGFP-F	GAATTTATATGTATACAACTGTACAGTCACGATCTCGTGAGAGATG	For maker gene
72EGFP-R	TAGCCTTCTTTGATATTCATCTTCCTGTGAGATCATGGCAGCTATA	
A137Rd-RA-F	AGCTGCCATGATCTCACAGGAAGATGAATATCAAAGAAGGCTA	For right arm
A137Rd-RA-R	TCGACGCGTCTGCAGAAGCTTCACAAATGGACAAACTTGG	
hTBK1-F	GAAGAGGAGACAACAACAAGA	qPCR for human *TBK1*
hTBK1-R	GGTAGTCCATAGGCATTAGAAG	
hGAPDH-F	GACACCCACTCCTCCACCTTT	qPCR for human *GAPDH*
hGAPDH-R	ACCACCCTGTTGCTGTAGCC	
hIFN-β-R	AATGCGGCGTCCTCCTTCT	qPCR for human *IFN-β*
hIFN-β-F	CAAATTGCTCTCCTGTTGTGCTTC	
hISG54-F	CTGCAACCATGAGTGAGAA	qPCR for human *ISG54*
hISG54-R	CCTTTGAGGTGCTTTAGATAG	
hISG56-F	TACAGCAACCATGAGTACAA	qPCR for human *ISG56*
hISG56-R	TCAGGTGTTTCACATAGGC	
pIFN-α-F	ATCCTTCTCTTCCTCCAG	qPCR for porcine *IFN-α*
pIFN-α-R	AGCATTTCTATGATGAACCA	
pIFN-β-F	GGCTGGAATGAAACCGTCAT	qPCR for porcine *IFN-β*
pIFN-β-R	TCCAGGATTGTCTCCAGGTCA	
pISG54-F	AAGACGGCAGAGAATGAA	qPCR for porcine *ISG54*
pISG54-R	GATAGGAGCAGACAAGGAA	
pISG56-F	TTAGAAAACAGGGTCTTGGAGGAG	qPCR for porcine *ISG56*
pISG56-R	CGTAAGGTAATACAGCCAGGCATA	
pGAPDH-F	GAAGGTCGGAGTGAACGGATTT	qPCR for porcine *GAPDH*
pGAPDH-R	TGGGTGGAATCATACTGGAACA	
Myc-A137R-F	ATGGAGGCCCGAATTCGGATGGAAGCAGTTCTTACC	For amplification of *A137R*
Myc-A137R-R	CGGCCGCGGTACCTCGAGTTAGCCTTCTTTGATATT	
GST-A137R-F	GGGCCCCTGGGATCCCCGGAATTCATGGAAGCAGTTCTTACC	For amplification of *A137R*
GST-A137R-R	TCAGTCACGATGCGGCCGCTCGAGTTAGCCTTCTTTGATATT	
Flag-cGAS-F	CAAGCTTGCGGCCGCGAATTCAATGGCGGCCCGGCGGGGAA	For amplification of *cGAS*
Flag-cGAS-R	CCTCTAGAGTCGACTGGTACCTCACCAAAAAACTGGAAA	
Flag-STING-F	CAAGCTTGCGGCCGCGAATTCAATGCCCTACTCCAGCCTGC	For amplification of *STING*
Flag-STING-R	CCTCTAGAGTCGACTGGTACCTCAGAAGATATCTGAGCGG	
Flag-TBK1-F	CAAGCTTGCGGCCGCGAATTCAATGCAGAGCACTTCTAATC	For amplification of *TBK1*
Flag-TBK1-R	CCTCTAGAGTCGACTGGTACCCTAAAGACAGTCAACATTG	
Flag-IRF3-F	CAAGCTTGCGGCCGCGAATTCAATGGGAACTCAGAAGCCT	For amplification of *IRF3*
Flag-IRF3-R	CCTCTAGAGTCGACTGGTACCCTAGAAATCCATGTCCTC	
Flag-IRF3-5D-F	GTGGACCTGCACATTGACAACGACCACCCGCTCGACCTCGACGACGACCAGTACAAGGC	For amplification of *IRF3-5D*
Flag-IRF3-5D-R	GCCTTGTACTGGTCGTCGTCGAGGTCGAGCGGGTGGTCGTTGTCAATGTGCAGGTCCAC	

a LA, left arm; RA, right arm; d, deletion; r, recombinant; F, forward; R, reverse; h, human; p, porcine.

### Dual-luciferase reporter (DLR) assays.

HEK293T cells were cotransfected with reporter plasmids (pIFN-β-Fluc or pISRE-Fluc [0.05 μg]) and pTK-Rluc (0.01 μg), along with or without the indicated expression plasmids, using X-tremeGENE HP (catalog no. 6366546001; Roche). After 24 h of transfection, the cells were washed with phosphate-buffered saline (PBS) three times. The passive lysis buffer was added for 20 min at 4°C with gentle shaking, and then the luciferase activities were determined using the DLR assay system (catalog no. E1910; Promega) according to the manufacturer’s instructions. The data are represented as the ratio of Fluc to Rluc.

### Generation of the ASFV-ΔA137R mutant.

To generate the ASFV-ΔA137R mutant by homologous recombination, the transfer vector was constructed, which harbors the *p72*p72 promoter-controlled *EGFP* gene and the left and right homologous arms of the *A137R* gene that are located in nucleotides (nt) 52692 to 54570 and 54962 to 55960, respectively, of the ASFV-WT genome. The nucleotides in the genome from positions 54571 to 54961 were replaced by the *p72*EGFP expression cassette. Briefly, the left and right homologous arms of the *A137R* gene with restriction enzyme sites were amplified using the gDNA of ASFV-WT as the template, and then fused to the *p72*EGFP expression cassette by overlapping PCR using the primers listed in Table 1, and the resulting fusion fragment was cloned into the pOK-12 vector. The transfer vector was sequenced and named pOK-p72-ΔA137R-EGFP.

The ASFV-ΔA137R mutant was generated by homologous recombination according to the previously described methods (61). Briefly, PAMs were seeded in 6-well plates coated with poly-L-lysine and incubated for 24 h at 37°C. The PAMs were transfected with 2 μg of transfer vector pOK-p72-ΔA137R-EGFP using X-tremeGENE HP for 16 h and then infected with ASFV-WT (MOI = 3). The recombinant viruses in PAMs were harvested until the EGFP expression, followed by purification using limiting dilution.

### NGS.

To confirm the accurate deletion of the *A137R* gene in the ASFV genome, the gDNA of ASFV-ΔA137R was extracted from the infected PAMs using the QIAamp blood mini kit (catalog no. 51104; Qiagen), and the full-length sequence of the ASFV-ΔA137R genome was determined by NGS as described previously (66).

### RNA-seq analysis.

PAMs were infected with ASFV-ΔA137R or ASFV-WT at an MOI of 1. At 4, 12, and 20 hpi, the cells were used to extract total RNAs by using TRIzol reagent (catalog no. 15596026; Invitrogen) according to the manufacturer’s instructions. RNA quantification and qualification were assessed using the RNA Nano 6000 assay kit of the Bioanalyzer 2100 system (Agilent Technologies, CA, USA). The mRNAs with poly(A) tails were enriched and purified from the total mRNAs by poly(T) oligonucleotide-coupled magnetic beads. RNA-seq libraries were prepared and assessed by AMPure XP system kit (Beckman Coulter, Beverly, USA) in the Agilent Bioanalyzer 2100 system. Subsequently, the RNA-seq libraries were sequenced on an Illumina Novaseq platform (Nova gene, China). KEGG enrichment analysis of DEGs was performed using Cluster Profiler (3.4.4).

### RNA extraction and RT-qPCR.

The total RNAs of the cells with indicated treatment or virus infection were extracted with the Simply P total RNA extraction kit (catalog no. BSC52M1; BioFlux) and transcribed into cDNA by FastKing gDNA Dispelling RT SuperMix (catalog no. KR118-02; Tiangen). The cDNAs from HEK293T cells or PAMs were used as templates for RT-qPCR using SYBR Premix *Ex Taq* (catalog no. RR390B; TaKaRa) to examine the mRNA level of human *IFN*-*β*, *ISG54*, *ISG56*, or *TBK1*, and porcine *IFN-α*, *IFN-β*, *ISG54*, or *ISG56*, respectively. The glyceraldehyde-3-phosphate dehydrogenase gene (*GAPDH*) was used as an internal reference, and all the primers are listed in Table 1.

### GST pulldown assay.

The GST pulldown assay was performed as described previously (65). In brief, HEK293T cells in 6-well plates were transfected with p3×Flag-cGAS, -STING, -TBK1, or -IRF3 (2 μg per well) for 48 h, and then the cells were lysed and collected. Subsequently, the purified GST or GST-pA137R protein was incubated with GST-agarose (catalog no. L00206; Genscript) for 12 h and centrifuged at 4°C, and then the agarose was washed twice with cold PBS at 4°C. Next, the Flag-cGAS, -STING, -TBK1, or -IRF3 expressed in HEK293T cells was incubated with the purified GST or GST-pA137R for 4 to 6 h and washed six times with cold PBS at 4°C. Finally, the bound proteins were analyzed by Western blotting using mouse anti-GST monoclonal antibody (MAb) (1:1,000) (catalog no. K200006M; Solarbio), mouse anti-Flag MAb (1:1,000) (catalog no. AE005; ABclonal), and mouse anti-GAPDH MAb (1:1,000) (catalog no. 60004-1-Ig; Proteintech).

### Co-IP assay.

PAMs were infected with ASFV-WT at an MOI of 1, and the lysates were collected at 48 hpi for the co-IP assay. Protein G agarose (catalog no. 11243233001; Roche) was incubated with the lysates and rabbit anti-pA137R PAbs or a rabbit immunoglobulin G (catalog no. ab190492; Abcam) (isotype control antibody). The bound proteins were subjected to Western blotting using rabbit anti-TBK1 (catalog no. 3504S; Cell Signaling Technology) and in-house mouse anti-pA137R PAbs.

### Laser confocal microscopy.

HEK293T cells were cotransfected with p3×Flag-TBK1 and pMyc-A137R or pCMV-Myc (0.5 μg each) for 36 h and fixed in 4% paraformaldehyde (catalog no. BL539A; Biosharp) for 30 min at room temperature. After being washed twice with PBS, the cells were permeabilized in 0.15% Triton X-100 (catalog no. 1139100; BioFroxx) for 15 min at room temperature. The cells were incubated with mouse anti-Myc MAb (1:500) (catalog no. M4439-100UL; Sigma-Aldrich) or rabbit anti-Flag PAbs (1:500) for 1.5 h at 37°C. Following incubation with Alexa Fluor 633-conjugated goat anti-mouse IgG (H+L) antibody (1:500) (catalog no. A21052; Invitrogen) or Alexa Fluor 488-conjugated donkey anti-rabbit IgG (H+L) antibody (1:500) (catalog no. A21206; Invitrogen) for 45 min, the cells were washed with PBS. Subsequently, the cells’ nuclei were stained with 4′,6-diamidino-2-phenylindole (DAPI) (1:1,000) (catalog no. D8417; Sigma-Aldrich) and observed by laser confocal microscopy (LSM880, Zeiss).

PAMs were infected with ASFV-WT (MOI = 1) for 24 h, and pA137R or TBK1 was examined with in-house mouse anti-pA137R (1:500) or rabbit anti-TBK1 PAbs (1:100) (catalog no. 703154; Invitrogen), respectively, as described above. The colocalization coefficients were determined using the ImageJ software.

### Cell viability assay.

Cell viability was determined by Cell Counting Kit-8 (catalog no. K1018; APExBIO) according to the manufacturer's instructions.

### Inhibitor treatment assay.

HEK293T cells were cotransfected p3×Flag-TBK1 with pMyc-A137R or pCMV-Myc for 24 h. Then the cells were treated with 20 μM proteasome inhibitor MG132 (catalog no. M8699-1MG; Sigma-Aldrich), 10 mM autophagosome inhibitor 3-MA (catalog no. HY-19312; Medchemexpress), or 100 nM BafA1 (catalog no. HY-100558; Medchemexpress) for 8 h, and the lysates were examined by Western blotting analysis.

### Nuclear translocation assay.

HEK293T cells were transfected with pMyc-A137R or pCMV-Myc (0.5 μg per well) for 24 h and then stimulated with SeV (MOI = 1) or poly(dA-dT) (2 μg/mL) for 8 h. The localization of IRF3 and pA137R was examined by laser confocal microscopy using rabbit anti-IRF3 PAbs (1:100) (catalog no. 11312-1-AP; Proteintech) and mouse anti-Myc MAb (1:500), respectivel, as described above.

### Statistical analysis.

All data were determined in triplicates and analyzed with SPSS 22.0 software (SPSS Software, Inc.). Student's *t* test was used to assess statistical significance. A *P* value of <0.05 was considered as significant.
